# Psychometric properties of the persian version of the Medical Artificial Intelligence Readiness Scale for Medical Students (MAIRS-MS)

**DOI:** 10.1186/s12909-023-04553-1

**Published:** 2023-08-15

**Authors:** AmirAli Moodi Ghalibaf, Maryam Moghadasin, Ali Emadzadeh, Haniye Mastour

**Affiliations:** 1 Student Research Committee, Faculty of Medicine, Birjand University of Medical Sciences, Birjand, Iran; 2 Department of Clinical Psychology, Faculty of Psychology and Education, Kharazmi University, Tehran, Iran; 3 Department of Medical Education, Faculty of Medicine, Mashhad University of Medical Sciences, Mashhad, Iran

**Keywords:** Artificial intelligence, Medical education, Medical students, Psychometric properties, Validity and reliability

## Abstract

**Introduction:**

There are numerous cases where artificial intelligence (AI) can be applied to improve the outcomes of medical education. The extent to which medical practitioners and students are ready to work and leverage this paradigm is unclear in Iran. This study investigated the psychometric properties of a Persian version of the Medical Artificial Intelligence Readiness Scale for Medical Students (MAIRS-MS) developed by Karaca, et al. in 2021. In future studies, the medical AI readiness for Iranian medical students could be investigated using this scale, and effective interventions might be planned and implemented according to the results.

**Methods:**

In this study, 502 medical students (mean age 22.66(± 2.767); 55% female) responded to the Persian questionnaire in an online survey. The original questionnaire was translated into Persian using a back translation procedure, and all participants completed the demographic component and the entire MAIRS-MS. Internal and external consistencies, factor analysis, construct validity, and confirmatory factor analysis were examined to analyze the collected data. A *P* ≤ 0.05 was considered as the level of statistical significance.

**Results:**

Four subscales emerged from the exploratory factor analysis (Cognition, Ability, Vision, and Ethics), and confirmatory factor analysis confirmed the four subscales. The Cronbach alpha value for internal consistency was 0.944 for the total scale and 0.886, 0.905, 0.865, and 0.856 for cognition, ability, vision, and ethics, respectively.

**Conclusions:**

The Persian version of MAIRS-MS was fairly equivalent to the original one regarding the conceptual and linguistic aspects. This study also confirmed the validity and reliability of the Persian version of MAIRS-MS. Therefore, the Persian version can be a suitable and brief instrument to assess Iranian Medical Students’ readiness for medical artificial intelligence.

## Introduction

The origin of artificial intelligence (AI) can be traced back to the 1950s when artificial neural networks were created [1]. The Accreditation Council for Graduate Medical Education (ACGME) has revised its internal medicine guidelines in light of AI’s potential to improve and reshape patient treatment and clinical management plans. In its 2019 guidelines, the American Medical Association (AMA) promoted AI implementation and discussed payment, regulation, deployment, and liability issues [2].

Numerous healthcare concerns can be solved globally using AI and its various applications, such as making and simplifying diagnoses, big-data analytics, administration, and overall clinical decision-making [3, 4]. Moreover, this approach enables physicians to provide diagnoses along with prognoses rapidly by minimizing the effort to analyze digital information [5, 6]. This is not a question of whether AI shall be applied to healthcare but rather when it will become a standard approach to optimize healthcare practice [4]. In this context, AI is expected to become essential to physicians’ professional practice [7–9].

AI is highly technical, requiring computer science knowledge and mathematical understanding as the basic foundations [10]. Training healthcare providers with adequate knowledge of AI presents unique challenges to clinical relevance, content selection, and the methods for teaching the concepts [11]. To achieve these, we need curriculum integration, performance assessments, and both interdisciplinary and research-based teaching guidelines [5, 12]. This process involves more than just learning technical skills associated with computer programming. Rather, one must have a comprehensive knowledge of basic, clinical and evidence-based medicine, together with biostatistics and data science [13]. In this context, it is critical for medical students to be provided with curricular and extracurricular opportunities to learn about technical limitations, clinical applications, and the ethical perspective of AI tools [14].

Therefore, it is imperative to integrate technical and non-technical principles of AI with medical students. However, the current medical schools’ teaching approaches are limited in their coverage of AI in their curricula [11]. Our review of the recent literature has uncovered the following important points on medical education: ***(a)*** the need for AI in the curricula of undergraduate medical education (UME), ***(b)*** recommending such a curricular delivery, ***(c)*** suggesting AI curriculum contents, particularly a focus on the ethical aspects of machine learning (ML) and AI, ***(d)*** integrating AI into UME curricula, and ***(e)*** the need for cultivating the uniquely human skills on empathy for patients in the face of AI-driven development and challenges [15].

These days, students in medical and dental schools have a good understanding of AI concepts, an optimistic attitude regarding it, and are interested in seeing AI incorporated into their curricula [16]. Thus, these students strongly believe that AI will profoundly impact medical education in the near future [17]. However, the curricula in medical schools do not currently have an AI component [3]. Although AI has numerous applications to improve the clinical management of patients, it remains unclear to what extent students and medical practitioners are using them in their education and later in medical practice [2, 4].

### Aim of the study

A psychometric measurement tool that is reliable and valid has been developed by Karaca, et al. [18]. to assess the medical students’ perceived readiness toward AI developments and their applications in medicine. Thus, this study aimed to investigate the psychometric properties of a Persian version of the Medical Artificial Intelligence Readiness Scale for Medical Students (MAIRS-MS) among Iranian medical students.

## Methods

### Sample

The sample population for the present study was selected from among the Iranian medical students at the Faculty of Medicine, Mashhad University of Medical Sciences, using convenience sampling. The students were informed through Email and social media messages, such as *WhatsApp* and *Telegram*, where the link to the MAIRS-MS survey was posted online. Two thousand three hundred thirty-eight students were eligible as the research population. The completed surveys were received from 502 medical students (275 female and 227 male).

### Demographic information

The study participants responded to questions related to demographics, such as gender, age, and the curriculum of the Doctor of Medicine (MD) program. The questions covered the basic science, preclinical, and clinical aspects of their MD curriculum in addition to those specific to MAIRS-MS.

**Medical Artificial Intelligence Readiness Scale for Medical Students (MAIRS-MS)**: MAIRS-MS was provided by Karaca, et al. [18]. with 22 questions and four categories of cognition, ability, vision, and ethics. The Cronbach alpha values were: 0.83 for cognition, 0.77 for ability, 0.723 for vision, and 0.632 for ethics categories. Also, the four-factor structure model fitted well with the respective indices (CFI = 0.938; TLI = 0.928; NNFI = 0.928; RMSEA = 0.094; and SRMR = 0.057).

### Translation process

Prior to the actual Persian translation, the consent of the original questionnaire’s author was obtained via Email. Initially, each item of the English questionnaire was translated into Persian by two academic experts in English translation. Secondly, another two bilingual university instructors examined each of the translated items in terms of meaning, accuracy, wording, spelling, and grammar. Then, a back translation to English and a comparison of the original questionnaire with the back-translated one were made. As a result of their suggestions and feedback, necessary revisions were made to the survey questions. Thirdly, five faculty members specializing in AI checked each of the items to ensure that the exact meaning was achieved by translation. All of these individuals were fluent in both Persian and English languages. Based on subsequent feedback from experts, the wording of seven questions was revised. Finally, the final Persian version of the questionnaire was approved, consisting of 22 questions, each on a 5-point Likert scale (1 = strongly disagree to 5 = strongly agree).

### Data Analysis

To conduct data analysis, the steps were carried out as follows.

1. Exploratory factor analysis was led by cross-validation for half of the sample (n = 251) using principal axis factoring with varimax rotation [19, 20]. The following criteria were applied to extract the factors: ***(a)*** one eigenvalue criterion based on Kairs and Guttman [21, 22], ***(b)*** parallel analysis and Velicer’s MAP test based on Hayton [23, 24], and ***(c)*** the scree test proposed by Cattell [25].
2. Confirmatory factor analysis was performed on the sample’s other half (n = 251) to cross-validate the analysis. The good fit indices examined in this study were Chi-square, Chi-square/df, root mean square error of approximation, the goodness of fit index, comparative fit index, normed fit index, non-normed fit index, adjusted goodness of fit index, incremental fit index, relative fit index, and standardized root mean square residual.
3. Each dimension’s internal consistency reliability coefficient (Cronbach’s alpha and composite reliability) was calculated.
4. The intra-class correlation coefficient was conducted to determine inter-rater reliability.

SPSS-23 was used to calculate exploratory factor analysis, internal consistency, and stability of the questionnaire. LISREL-8.70 software was also used to calculate the confirmatory factor analysis.

## Results

### Descriptive statistics

Out of 502 medical students aged 17 to 36, the mean age was 22.66(± 2.767), with 55% of the sample being female. The participants in each curricular phase of the MD program consisted of basic science (146 (29%)), preclinical (115(23%)), and clinical (241 (48%)). The mean, standard deviation, maximum and minimum, skewness, and kurtosis of scores obtained from the collected data are shown in Table 1. Since the sample size was larger than 300 individuals, Kolmogorov–Smirnov and Shapiro-Wilk’s tests might be unreliable. Both the skew and kurtosis could be analyzed by descriptive statistics. The acceptable values for skewness fell between ˗ 3 and + 3, and the kurtosis was appropriate from ˗ 10 to + 10 when structural equation modeling was introduced [26]. In this study, the skew and kurtosis were less than the absolute value of one. Therefore, the results indicated that the data distribution was normal.

**Table 1:** Descriptive statistics of MAIRS-MS scores

Categories	Mean	SD	Min	Max	Skewness	Kurtosis
Total score	63.09	15.92	22	110	-0.184	-0.113
Cognition	20.42	6.11	8	40	0.050	-0.157
Ability	23.64	6.82	8	40	-0.291	-0.474
Vision	8.74	2.97	3	15	-0.220	-0.678
Ethics	10.30	2.74	3	15	-0.666	0.187

Note: SD = Standard Deviation

### Exploratory factor analysis

**Fig. 1:**
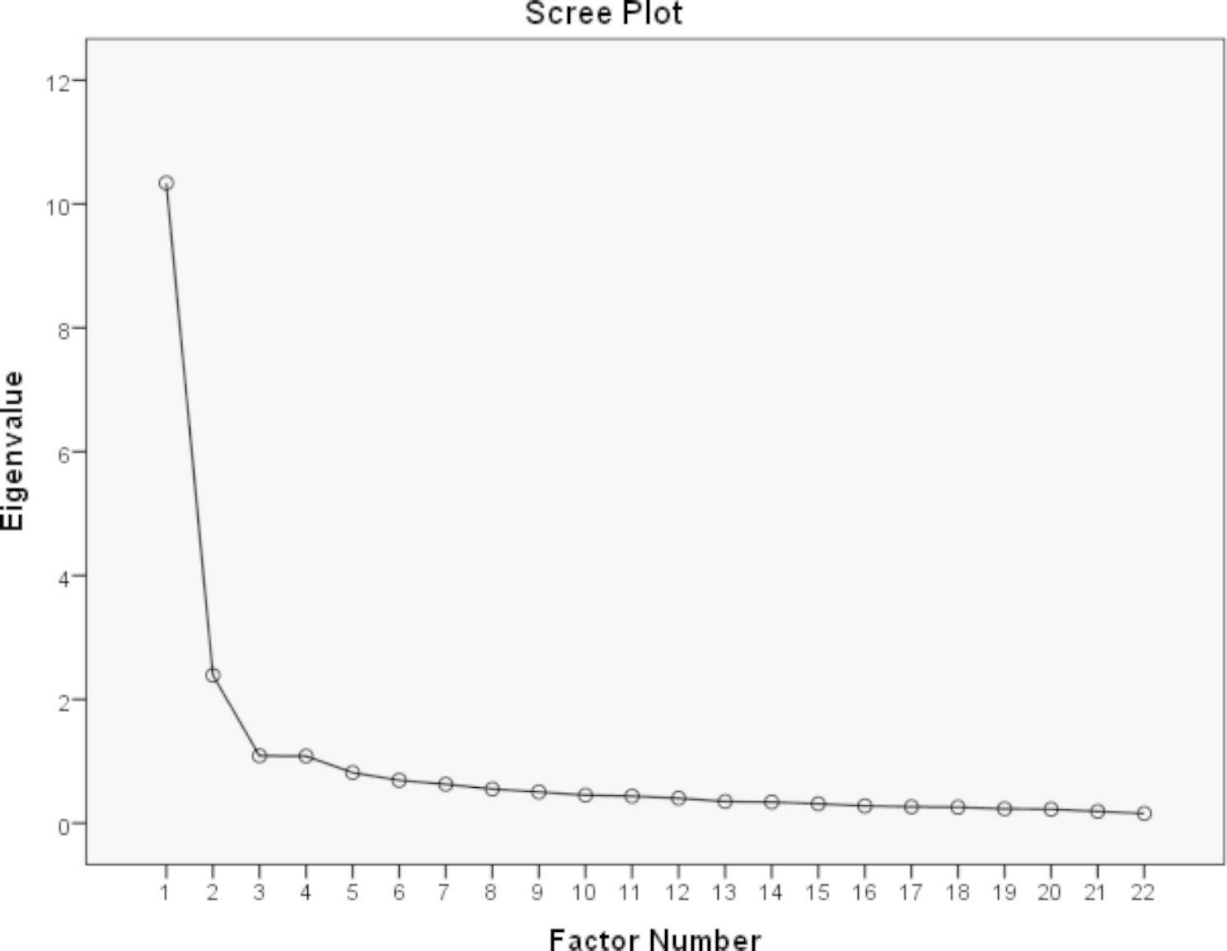
Scree plot of the principal axis factoring

The results of the exploratory factor analysis of the Persian version of MAIRS-MS are shown in Table 2. Principal axis factoring was performed for extraction factors, resulting in four factors consistent with the original MAIRS-MS [18]. Also, the number of factors by parallel analysis and Velicer’s MAP test was equal to four. These factors accounted for 63% of the common variance, which was acceptable considering that the expected variance in social science studies is known to be 60% [27, 28]. Out of 63% of the model’s total variance, 19% was explained by the first factor (Cognition). The variances explained by factors 2, 3, and 4 were 18%, 14%, and 12%, respectively. Table 2 shows the relationship between the factors and variables, i.e., questions. All values in Table 2 are above 0.40, indicating a strong relationship between the questions and the respective factors [29]. In addition, the Kaiser-Meyer-Olkin test result was 0.945 (i.e., above 0.60) and the Bartlett test result was satisfactory (X^2^ = 7478.39; df = 231; *P* ≤ 0.00001). Also, the scree diagram shows the four mentioned factors with eigenvalues greater than one (Fig. 1).

**Table 2:** Exploratory factor analysis with principal axis factoring of MAIRS-MS (Varimax rotation transformation including factors with eigenvalue of one or more)

Questions	Factor 1 (Cognition)	Factor 2 (Ability)	Factor 3 (Ethics)	Factor 4 (Vision)
1. I can define the basic concepts of data science.	**0.561** ^*^	0.099	0.081	0.113
2. I can define the basic concepts of statistics.	**0.475** ^*^	0.141	0.100	0.111
3. I can explain how AI systems are trained.	**0.761** ^*^	0.207	0.035	0.233
4. I can define the basic concepts and terminology of AI.	**0.807** ^*^	0.208	0.020	0.183
5. I can properly analyze the data obtained by AI in healthcare.	**0.579** ^*^	0.250	0.169	0.207
6. I can differentiate the functions and features of AI related tools and applications.	**0.602** ^*^	0.280	0.074	0.230
7. I can organize workflows compatible with AI.	**0.585** ^*^	0.277	0.057	0.199
8. I can express the importance of data collection, analysis, evaluation and safety; for the development of AI in healthcare.	**0.409** ^*^	0.210	0.266	0.302
9. I can harness AI-based information combined with my professional knowledge.	0.297	**0.653** ^*^	0.256	0.217
10. I can use AI technologies effectively and efficiently in healthcare delivery.	0.187	**0.753** ^*^	0.285	0.234
11. I can use artificial intelligence applications in accordance with its purpose.	0.181	**0.714** ^*^	0.223	0.240
12. I can access, evaluate, use, share and create new knowledge using information and communication technologies.	0.128	**0.542** ^*^	0.271	0.173
13. I can explain how AI applications offer a solution to which problem in healthcare.	0.220	**0.484** ^*^	0.161	0.295
14. I find valuable to use AI for education, service and research purposes.	-0.007	**0.622** ^*^	0.179	0.131
15. I can explain the AI applications used in healthcare services to the patient.	0.201	**0.414** ^*^	0.133	0.263
16. I can choose proper AI application for the problem encountered in healthcare.	0.287	**0.492** ^*^	0.269	0.208
17. I can explain the limitations of AI technology.	0.226	0.271	0.173	**0.684** ^*^
18. I can explain the strengths and weaknesses of AI technology.	0.254	0.247	0.295	**0.743** ^*^
19. I can foresee the opportunities and threats that AI technology can create.	0.239	0.198	0.225	**0.643** ^*^
20. I can use health data in accordance with legal and ethical norms.	0.139	0.239	**0.661** ^*^	0.236
21. I can conduct under ethical principles while using AI technologies.	0.086	0.166	**0.862** ^*^	0.128
22. I can follow legal regulations regarding the use of AI technologies in healthcare.	0.091	0.146	**0.762** ^*^	0.193
**Eigenvalues**	**18.999** ^*^	**17.813** ^*^	**14.056** ^*^	**11.932** ^*^
**Explained variance** (%)	**18.999** ^*^	**36.812** ^*^	**50.868** ^*^	**62.80** ^*^

*Loadings with absolute values of ≥ 0.40 are shown in bold

### Confirmatory factor analysis

**Fig. 2:**
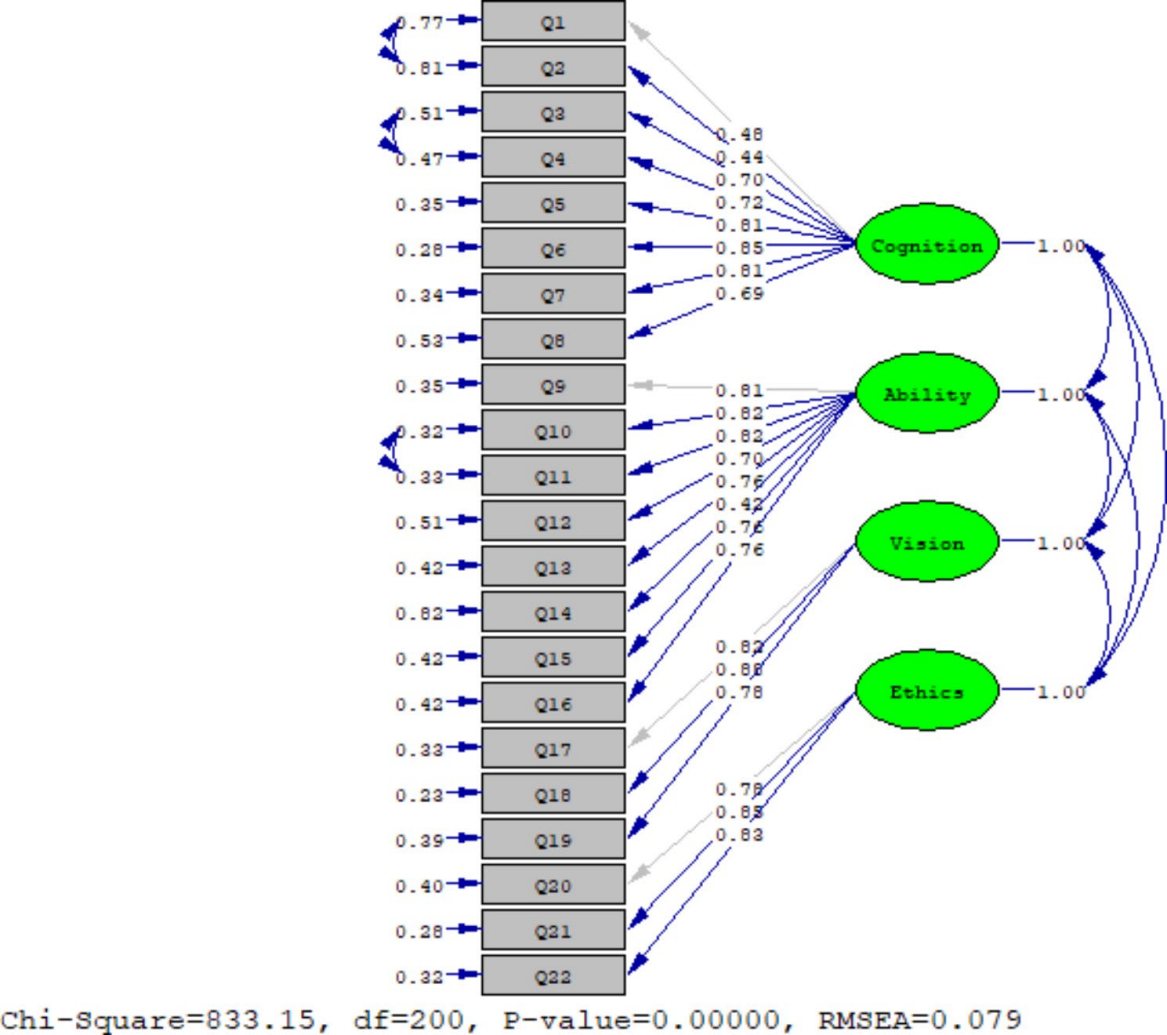
Standardized coefficients for the four-factor model of MAIRS-MS

In order to evaluate the conceptual model of the original version of the questionnaire, confirmatory factor analysis was applied using LISREL-8.70 software. This was performed on the sample’s second half (n = 251) to cross-validate the analysis. The estimation method in the confirmatory factor analysis was the maximal likelihood, assuming that the observed indicators follow a continuous and multivariate normal distribution. As shown in Fig. 2, the relationship of all extracted factors with the observed variables (i.e., questions) was desirable (standardized factor loading greater than 0.40) [29]. Also, the fit indices (X^2^ = 833.15; df = 200; *P* ≤ 0.000001; RMSEA = 0.079) revealed an acceptable fit of the model with respect to the data.

Since values above 0.90 for the variables were acceptable, the values, as shown in Table 3, indicated a relatively good fit [30, 31]. Also, first-order confirmatory factor analysis was used to evaluate the construct validity of the questions. The unstandardized factor loading of questions, R-squared (R^2^), and average variance extracted (AVE) were calculated for each factor (Table 4). The results showed that according to the amount of factor loading obtained, which is more than 0.40 and are at a significant level of less than 0.05 (*P* ≤ 0.05) (all T values are greater than 1.96). Therefore, the finding indicated that the construct validity for all questions was established. In addition, the results showed that the AVE index was greater than 0.50. The AVE index represented the average variance extracted for each factor by its questions. Thus, the larger the index, the greater the fit [32, 33].

To modify the original model, the error covariance between questions 1 and 2 on the *Cognition* subscale (decreased Chi-square = 67.02), the error covariance between questions 3 and 4 on the *Cognition* subscale (decreased Chi-square = 51.25), and the error covariance between questions 10 and 11 on the *Ability* subscale (decreased Chi-square = 42.33) was released due to the correlation between the error covariance in these items. Modifying the original model improved the RMSEA by approximately 0.02 (decreased from 0.094 to 0.079).

**Table 3:** Model fit indices determined via confirmatory factor analysis

Fitness indices	Observed value	Expected value
Chi-square	833.15	-
df	200	-
Chi-square/df	4.16	≤ 5
RMSEA 90% CI RMSEA	0.079 0.074–0.085	≤ 0.08
GFI	0.93	≥ 0.90
CFI	0.92	≥ 0.90
NFI	0.90	≥ 0.90
NNFI	0.91	≥ 0.90
AGFI	0.92	≥ 0.90
IFI	0.92	≥ 0.90
RFI	0.93	≥ 0.90
TLI	0.92	≥ 0.90
SRMR	0.048	≤ 0.05

Note: RMSEA = Root Mean Square Error of Approximation; GFI = Goodness of Fit Index; CFI = Comparative Fit Index; NFI = Normed Fit Index; NNFI = Non-Normed Fit Index; AGFI = Adjusted Goodness of Fit Index; IFI = Incremental Fit Index; RFI = Relative Fit Index; TLI = Tucker–Lewis Index; SRMT = Standardized Root Mean Square Residual

**Table 4:** Factor loading, T value for each question, and AVE for each factor

Factors	Unstandardized factor loading	T value	R^2^	AVE
Factor 1: (Cognition)				
Question 1	1.00	10.22	0.43	**0.56**
Question 2	1.24	9.72	0.49	
Question 3	1.53	10.26	0.51	
Question 4	1.51	10.41	0.53	
Question 5	1.75	10.90	0.65	
Question 6	1.77	11.09	0.72	
Question 7	1.63	10.92	0.66	
Question 8	1.70	10.17	0.47	
**Factor 2: (Ability)**				
Question 9	1.00	21.19	0.65	**0.60**
Question 10	1.14	21.17	0.68	
Question 11	1.14	21.00	0.67	
Question 12	1.13	17.12	0.59	
Question 13	1.12	17.11	0.58	
Question 14	1.13	17.12	0.48	
Question 15	1.15	19.04	0.58	
Question 16	1.15	19.04	0.58	
**Factor 3: (Vision)**				
Question 17	1.00	21.87	0.67	**0.68**
Question 18	1.14	22.07	0.77	
Question 19	1.01	19.29	0.61	
**Factor 4: (Ethics)**				
Question 20	1.00	18.22	0.60	**0.67**
Question 21	1.05	18.93	0.72	
Question 22	1.05	18.54	0.68	

**Reliability**: Cronbach alpha was used to investigate the internal consistency of the MAIRS-MS questions. The respective values for the *Cognition, Ability, Vision*, and *Ethics* subscales were: 0.886, 0.905, 0.865, and 0.856. Also, the Cronbach alpha value for the total scale was 0.944. These values, which were similar to those of the original questionnaire, demonstrated the data’s acceptable internal consistency and reliability. In addition, the composite reliability coefficients for the subscales were 0.972 (Cognition), 0.884 (Ability), 0.879 (Vision), and 0.894 (Ethics). The composite reliability coefficient for the total scales was 0.981. Finally, the inter-rater reliability was determined for each of the subscales as follows: 0.703 (Cognition), 0.753 (Ability), 0.979 (Vision), and 0.715 (Ethics). The Cronbach alpha intra-class correlation coefficient for the total scale was 0.871.

## Discussion

AI has transformed healthcare procedures [34] and is viewed and expected to reshape the learning process in medical education and healthcare services as we move into a new era. In addition, natural language processing techniques and large language models have significantly advanced AI applications [35]. However, AI shall not replace the roles of physicians and professors in today’s academia; rather, it may modify the roles [36]. Due to these developments, the current knowledge of medical students and practicing physicians on AI is likely to foster such an evolution [37, 38]. Furthermore, medical education faces pedagogical issues on how AI should be introduced into medical curricula to be maximally effective but not destructive. This is still a controversial subject in the delivery of healthcare to the public [7]. Despite the positive aspects of AI, it may not fill all aspects of the emotions, empathy, and direct communication appropriate to the healthcare system. Thus, many unclear areas exist regarding the application of AI in medical education and the professional setting [18].

The present study was conducted to determine the psychometric properties of the Persian version of MAIRS-MS. The scale consisted of 22 items, and exploratory factor analysis revealed that the MAIRS-MS had four factors: cognition, ability, vision, and ethics. The preliminary results showed high consistency of the Persian version with the original questionnaire with respect to each of the four factors. Our detailed statistical analyses indicated an acceptable validity of all questions on the Persian version of the MAIRS-MS. The reliability analyses of that version led to high values representing its reliability. Hence, this study established the validity and reliability of the Persian version of MAIRS-MS.

Despite the lack of explicit opinions on the effects of AI on healthcare delivery and/or medical education, several studies have developed scales to evaluate the attitude, knowledge, and readiness of healthcare professionals and medical students on the introduction of AI in medicine and medical education. MAIRS-MS has been developed as a 4-subscale tool to assess cognition, ability, vision, and ethics among the currently available scales. The original version has been evaluated as a highly valid and reliable tool for monitoring medical students’ perceived readiness toward AI technologies and applications [18]. In this regard, a Chinese scale has also been developed by Li, et al., which evaluates the personal relevance of medical AI, subjective norms and self-efficacy in learning AI, basic knowledge, behavioral intention, and the actual learning of AI. Despite the 25-item scale being seen as a valid and reliable tool in Chinese, its main aim was to understand medical students’ perceptions of and behavioral intentions toward learning AI, which revealed some differences compared to that of the original English MAIRS-MS [39].

Further, Boillat, et al. developed a questionnaire to capture medical students’ and physicians’ familiarity with AI in medicine and the challenges, barriers, and potential risks linked to the democratization of this new paradigm. The scale consisted of 31 items under five factors, such as familiarity with medical AI, education and training, challenges and barriers in medical AI and its implementation, as well as the risks linked to medical AI [4]. Although the existing scales have some overlapping aspects, they are not identical. The differences among the various questionnaires may cover the gaps and utilize appropriate scales in specific populations to achieve particular goals.

Due to the impact of AI in medicine and medical education, several studies have evaluated medical students’ opinions on AI aiming at bringing further improvement to this method. Abid, et al. investigated Pakistanis medical students’ attitudes and readiness toward AI [40]. In the United Kingdom, Sit, et al. revealed that 89% of medical students believed that teaching in AI would be beneficial to their future medical practice. In this study, 78% of the respondents agreed that students should receive training on AI as part of their medical curricula [41]. Conversely, there are significant issues, such as knowledge, attitudes, and readiness about AI in some developing countries. Hamd, et al. study results showed a lack of education and training programs for AI implementation, and from their point of view, organizations were not well prepared and had to ensure readiness for AI [42]. In the United Arab Emirates, Boillat, et al. reported low familiarity with AI and called for specific training provided at medical schools and hospitals to ensure that they can leverage this new paradigm to improve upon their healthcare delivery and clinical outcomes [4]. It seems that the differences between the developed and developing countries are largely due to their curricular designs, especially concerning the role of AI or lack thereof. Therefore, it is recommended that medical schools consider mechanisms for knowledge sharing about AI and develop curricula to teach the use of AI tools as a competency [43]. In addition, medical students should be practically exposed to AI technology incorporations [44]. The effort to ensure professional and student readiness will improve AI integration in practice [42].

Finally, as a strong finding of the current study, it can be stated that we evaluated the psychometric properties of a novel and beneficial questionnaire among Iranian medical students and explored their readiness for AI in their curricula. In future studies, the student’s readiness for having AI components in the medical curricula will be further investigated using the translated scale developed in the current study, and its efficacy and potential will be assessed in greater detail and from different perspectives.

## Conclusions

The findings of this study revealed that the Persian MAIRS-MS was essentially equivalent in nature to the original one regarding the conceptual and linguistic aspects. This study also confirmed the validity and reliability of the Persian MAIRS-MS. Therefore, the Persian version can be a suitable and brief instrument to assess the readiness of Iranian medical students with respect to the inclusion of AI in their medical education and its impact on their future medical practice.

## Data Availability

The datasets used and/or analyzed during the current study are available from the corresponding author upon reasonable request.

## Abbreviations

MAIRS-MS: Medical Artificial Intelligence Readiness Scale for Medical Students
AI: Artificial Intelligence
ACGME: Accreditation Council for Graduate Medical Education
AMA: American Medical Association
UME: Undergraduate Medical Education
ML: Machine Learning
MD: Doctor of Medicine
SD: Standard Deviation
AVE: Average Variance Extracted
RMSEA: Root Mean Square Error of Approximation
GFI: Goodness of Fit Index
CFI: Comparative Fit Index
NFI: Normed Fit Index
NNFI: Non-Normed Fit Index
AGFI: Adjusted Goodness of Fit Index
IFI: Incremental Fit Index
RFI: Relative Fit Index
TLI: Tucker–Lewis Index
SRMT: Standardized Root Mean Square Residual
